# A total-internal-reflection-based Fabry–Pérot resonator for ultra-sensitive wideband ultrasound and photoacoustic applications

**DOI:** 10.1016/j.pacs.2023.100466

**Published:** 2023-02-24

**Authors:** Xiaoping Jiang, Mengqi Shen, Daniel Pak-Kong Lun, Wen Chen, Michael G. Somekh

**Affiliations:** a Department of Electronic and Information Engineering, The Hong Kong Polytechnic University, Hong Kong, China; b Nanophotonics Research Centre, Shenzhen Key Laboratory of Micro-Scale Optical Information Technology, Shenzhen University, Shenzhen 518060, Guangdong, China; c Guangdong Laboratory of Machine Perception and Intelligent Computing, The Faculty of Engineering, Shenzhen MSU-BIT University, Shenzhen 518172, Guangdong, China; d The Faculty of Engineering, University of Nottingham, Nottingham, UK

**Keywords:** Fabry–Pérot, Photoacoustic, Ultrasound, Transducer, Sensing, Ultra-sensitive, Wideband

## Abstract

In photoacoustic and ultrasound imaging, optical transducers offer a unique potential to provide higher responsivity, wider bandwidths, and greatly reduced electrical and acoustic impedance mismatch when compared with piezoelectric transducers. In this paper, we propose a total-internal-reflection-based Fabry–Pérot resonator composed of a 12-nm-thick gold layer and a dielectric resonant cavity. The resonator uses the same Kretschmann configuration as surface plasmon resonators (SPR). The resonators were analyzed both theoretically and experimentally. The experimental results were compared with those for an SPR for benchmarking. The 1.9-μm-thick-PMMA- and 3.4-μm-thick-PDMS-based resonators demonstrated responsivities of 3.6- and 30-fold improvements compared with the SPR, respectively. The measured bandwidths for the PMMA, PDMS devices are 110 MHz and 75 MHz, respectively. Single-shot sensitivity of 160 Pa is obtained for the PDMS device. The results indicate that, with the proposed resonator in imaging applications, sensitivity and the signal-to-noise ratio can be improved significantly without compromising the bandwidth.

## 1 Introduction

Photoacoustic and ultrasound imaging techniques have been developing rapidly over the past two decades for use as nondestructive imaging in industrial, clinical, and biomedical diagnosis applications [1], [2], [3], [4]. The main focus of the research into these techniques has been to improve their sensitivity and bandwidth to achieve higher resolution and image quality [5], [6], [7]. The piezoelectric effect has been used widely in the detection of ultrasound by using materials that have no inversion symmetry to convert mechanical vibrations into electrical energy [8]. Although it has long been possible to produce piezoelectric transducers with high center frequencies ranging up to the gigahertz band, there are several limitations to this approach: (i) the bandwidth is normally narrow, which restricts the ability to resolve features of different scales, particularly along the direction of propagation; (ii) meticulous control of electrical impedance matching is required to ensure optimal sensitivity and bandwidth; (iii) the bulky size and optical opacity of the transducers further hinder integration with other imaging modalities; and (iv) the fabrication of high-frequency transducers becomes increasingly demanding as the center frequency increases [9], [10].

Optics has emerged as a suitable approach to realizing ultrasound generation and detection with high bandwidths, optical transparency, and device miniaturization capabilities [9]. Optical sensors can eliminate the requirement for electrical impedance matching faced by conventional piezoelectric methods, while optical materials are available with acoustic impedances which are close to that of water, therefore allowing the acoustic impedance mismatch problems encountered by piezoelectric transducers to be ameliorated [10].

The two mechanisms used in optical sensors to enable ultrasound detection are (i) detection of the refractive index change in the sensing region induced by acoustic pressure, i.e., the acousto-optic effect, and (ii) detection of the pressure-induced cavity length change in a resonator, e.g., a Fabry–Pérot (FP) resonator [11], [12], [13], [14]. Conventional refractive index sensors, such as prism-based surface plasmon resonance (SPR) sensors, critical-angle-based optical surface wave (OSW) sensors and microring resonators, can be converted into acoustic sensors [15], [16], [17], [18], [19]. High bandwidth has been predicted up to gigahertz regime [9], [15]. Bandwidth over 170 MHz have been experimentally demonstrated with both critical-angle-based methods and SPR sensors [19], [20]. However, given that the acousto-optic coefficient of water is $1.35\times 10^{-10}\mathrm{RIU}∙{\mathrm{Pa}}^{-1}$, sensitivity performance of these devices is limited because of the relatively small refractive index change induced [7], [21].

On the other hand, the FP resonator structure is well-established in photoacoustic sensing and imaging applications and consists of an optical cavity made from a dielectric spacer sandwiched between two reflecting mirrors [11], [22]. The resonance condition of an FP mode follows the round-trip resonance conditions given [23].

(1) $$2k_{z}d+\phi_{1}+\phi_{2}=2m\pi ,m=0\left(\mathrm{for}\;\mathrm{TE}\right),1,2,3,...$$

where $k_{z}$=$\frac{2\pi }{\lambda }n_{s}\cos\theta$ denotes the *z*-component of the wave vector inside the spacer, d denotes the spacer thickness, i.e., the cavity length, $n_{s}$denotes the refractive index of the spacer, $\phi_{1}$ and $\phi_{2}$ denote the phase changes at the upper and lower interfaces of the cavity, respectively, m is a non-negative integer denoting FP orders, where *m* can be 0 with TE polarization (but not TM), λ is the incident wavelength, and θ is the propagation angle inside the spacer. An incident acoustic wave causes the separation of the dielectric reflectors to change by a small amount, which then results in a change in the optical reflectivity near resonance. The stress in the layer caused by the acoustic wave also induces a change in the refractive index, i.e., the acousto-optic effect, but this effect can normally be ignored [24], [25].

There have been extensive research interests in planar FP transducers. Metal was usually used in conventional FP transducers as the reflecting mirrors. However, a thick layer of metal makes the sensors opaque and unable to incorporate with other imaging modalities. Transparent structures have been used as the reflecting mirrors, e.g., Bragg reflectors and metasurfaces, which can enhance the capability of integration and allow backward-mode photoacoustic imaging [26], [27], [28]. FP sensors have also been made, which consist of polymer substrates and soft dielectric mirrors, thus reducing the acoustic impedance mismatch and providing a broader bandwidth compared with hard dielectric mirrors, providing a flatter and more broadband frequency response [11], [29], [30]. Other than planar FP transducers, plano-concave FP transducers that were fabricated with cured free-standing spherical caps and two eight-layered dielectric mirrors on a fiber tip have been proposed for high sensitivity photoacoustic imaging applications [26]. The abovementioned dielectric mirror contains a stack of 8 layers of λ/4 thickness. This can increase sensitivity and improve optical transparency, albeit at the price of the fabrication complexity and time.

In evaluation of the optical detection mechanisms for ultrasound, three parameters are most important, i.e., bandwidth, responsivity and sensitivity. Responsivity is defined as reflectivity change with respect to incident pressure. Sensitivity usually refers to the noise equivalent pressure (NEP), which is related to both the noise level of the experiment system and the responsivity of the transducer.

In terms of sensitivity and responsivity, the performance of the FP sensors is considerably better than that of refractive index sensing based mechanism within the bandwidth. The performance is even more pronounced as the thickness of the cavity increases [11]. However, the transducer mechanism of FP possesses an intrinsic trade-off between the bandwidth and sensitivity [26] since the sensitivity of FP sensors rolls off when the acoustic wavelength is of the same order as the cavity dimensions. This aspect is discussed in greater detail in Section 2. The bandwidth for refractive index sensing based mechanisms is only related to the penetration depth of the evanescent field, and the sensitivity is related to the field confinement on the surface, which is considered in Appendix 3.

**Fig. 1:**
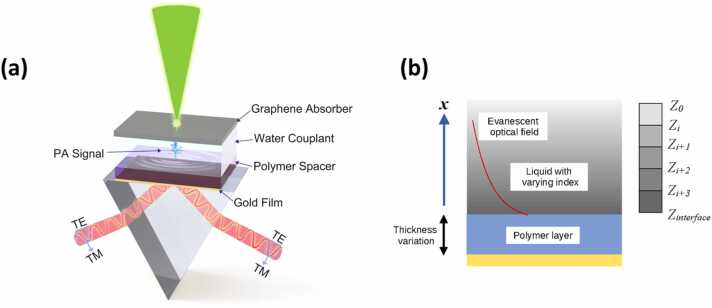
(a) Layout of the proposed total-internal-reflection-based Fabry–Pérot structure and the prism-based Kretschmann configuration. Also shown is the photoacoustic generation system used for system evaluation. PA: photoacoustic; TM: transverse magnetic; TE: transverse electric. (b) An illustration of the transduction mechanisms of the TIR-FP showing the thickness variation and the sensing of refractive index changes in the liquid. Note the extent of the evanescent field is exaggerated for clarity.

In this paper, we show that the use of an open resonator structure confers advantages for both the refractive index sensing mechanism (giving an improved bandwidth) and the FP mechanism (giving greater sensitivity). Fig. 1(a) shows a layout of the proposed sample structure and the conventional Kretschmann configuration used for interrogation of the device. The structure consists of two layers, i.e., a metal layer and a homogeneous dielectric spacer acting as the FP cavity. Fig. 1(b) shows an illustration of different transduction mechanisms that operate in the proposed structure where both thickness variation and pressure induced refractive index variation can be detected.

In this paper, we present a total-internal-reflection-based Fabry–Pérot resonator (TIR-FP) structure operating around the optical wavelength of 1550 nm and assess the device both theoretically and experimentally. The optimum thickness is determined for each layer by optimizing the responsivity via simulations. The structure can be fabricated on top of a conventional coverslip. Compared with the conventional FP structure, the proposed structure not only provides better acoustic impedance matching, therefore giving higher responsivity, but also offers a wider bandwidth. Moreover, the open resonator structure gives a wider choice of the resonator material since there is no requirement for the spacer to support a reflecting structure. The simpler fabrication process promises better yield and exceptionally inexpensive fabrication.

The modes of the designed TIR-FP structure are excited using a prism-based Kretschmann configuration, as shown in Fig. 1, which is the same as the configuration used in a conventional SPR sensor, for this reason, comparison of responsivity for the current system and SPR are presented in Appendix 4. Similar to a conventional FP resonator, two reflecting interfaces exist in the TIR-FP. The metal layer operates as a partially reflecting mirror to control the coupling into the resonator. When the angle of incidence exceeds the critical angle of the spacer/water interface, the interface then acts as a reflecting mirror. This greatly simplifies the fabrication requirements and means that there is no need to fabricate a reflecting layer on the polymer surface. This has major consequences, because soft polymers, such as polydimethylsiloxane (PDMS), are very attractive for acoustic transducer formation, but it can be difficult to form a reflecting layer coating on top of these materials as they are highly prone to cracking. These materials can be used in the proposed structure because there is no need to form another coating on top of them. The greater compliance of these materials means that for a given thickness, the resulting resonator is much more sensitive. The corollary is that for a given sensitivity, the smaller resonator thickness confers a greater bandwidth. These effects are discussed in Section 2 and experimental results are provided to show that a polymethyl methacrylate (PMMA)-based TIR-FP provides a 3.6-fold improvement in responsivity when compared with that of an SPR sensor, whereas the PDMS-based TIR-FP gives a 30-fold improvement. With regard to bandwidth, the PMMA, PDMS, and SPR devices provided bandwidths of 110 MHz, 75 MHz, and 113 MHz, respectively. Therefore, the TIR-FP is a convenient and immediate replacement resonator structure that will give orders of magnitude improvements over SPR structures in various ultrasound and photoacoustic sensing and imaging applications [31], [32], [33].

This paper is divided into four parts. Section 2 provides a theoretical analysis and offers intuitive analysis where fundamental working principles of the proposed structure are discussed and different operating principles are described, along with their advantages and disadvantages. Section 3 presents and discusses the experimental results for responsivity and bandwidth and offers a direct comparison with the corresponding properties of conventional SPR acoustic sensors. Sensitivity is also discussed on the basis of the noise equivalent pressure. Finally, conclusions are drawn and potential future enhancements are discussed in Section 4.

## 2 Theoretical analysis of working principles

As mentioned in the Introduction, the proposed resonator operates as an FP cavity, with the gold layer acting as a partial reflector, and the interfaces between the spacer and the water form a total internal reflection layer that acts as a perfect reflector. Sound waves perturb this cavity in two different ways: (i) by changing the phase of the reflection coefficient at the spacer/water interface, and (ii) by changing the cavity dimensions.

### 2.1 Mode analysis

**Fig. 2:**
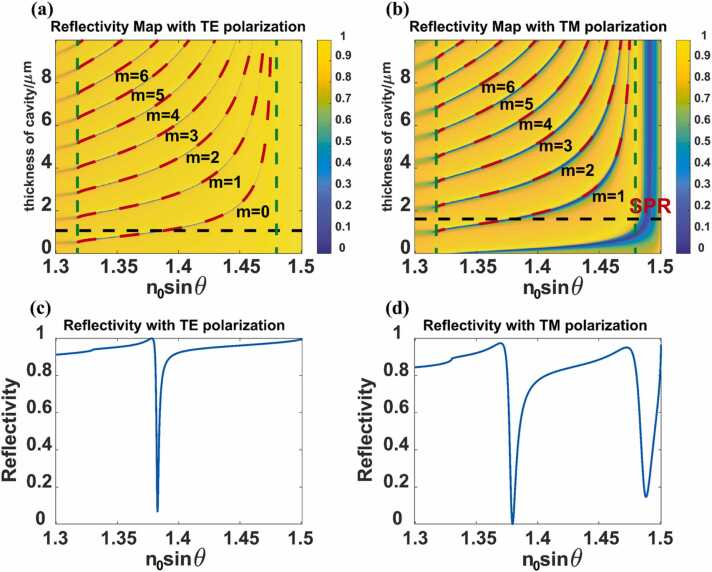
Reflectivity maps for PMMA-based structure varying $n_{0}\sin\theta$ and cavity thickness values for (a) TE and (b) TM polarizations. (c) Reflectivity with the 1-µm-thick cavity and the TE polarization. (d) Corresponding reflectivity with the 1.6-µm-thick cavity and the TM polarization.

First, we consider the cavity without perturbation. Under the round-trip resonance conditions described in Eq. (1), $\phi_{1}$ and $\phi_{2}$, which are the phase shifts at the top and bottom of the cavity, can be calculated as the phase angles of the reflection coefficient at the interface between the cavity and the water and that at the interface between the cavity and the gold layer, respectively. Fig. 2(a) and (b) show reflectivity maps with TE and TM polarizations, respectively, obtained at an interrogation wavelength of 1550 nm by varying the FP cavity thickness and the angle of incidence from the prism. The reflectivity is plotted against $n_{0}\sin\theta$ rather than against sinθ alone, this means that these plots are independent of the choice of prism material. The gold layer thicknesses are 12 nm and 30 nm for the TE and TM polarizations, respectively. In the calculations, the refractive index values used for glass ($n_{0}$), the gold layer ($n_{1}$), the FP cavity ($n_{2}$), and water ($n_{\mathrm{water}}$) at 1550 nm were 1.501, 0.84991 + 10.925i, 1.4771, and 1.3180, respectively [34]. The value of $n_{0}\sin\theta$ for the critical angle of the glass-water interface was 1.3180, and that for the glass-PMMA interface was 1.4771. The red dashed lines in Fig. 2(a) and (b) represent the *k*-vector positions of the modes when calculated using the round-trip resonance conditions alone. The green dashed lines represent $n_{0}\sin\theta$ of the two critical angles, represent in the operating region of the device. For the TE mode, the zeroth order exists because the sum of $\phi_{1}$ and $\phi_{2}$ within the operating regime is negative. Fig. 2(c) and (d) show the reflectivity characteristics with the 1μm cavity thickness for the TE polarization and the 1.6μm cavity thickness for the TM polarization, respectively. The thicknesses were selected to allow FP resonances to be compared at the same $n_{0}\sin\theta$ because both the resonance linewidth and the responsivity are dependent on $n_{0}\sin\theta$. It can be confirmed that the operating modes are FP modes, because the red dashed lines that represent the dip positions calculated based on the roundtrip resonance conditions overlap perfectly with the dips calculated via Fresnel calculations.

### 2.2 Detection mechanisms

We now consider two principal detection mechanisms. These mechanisms will be considered to be independent, which is a valid simplification for ultrasound signals at practical levels.

In SPR sensors, a change in the refractive index of the liquid modifies the resonance conditions at the gold-water interface; therefore, for an optical beam at a fixed angle of incidence, the small change in the resonance condition induced by the refractive index perturbation will result in a change in the optical reflectivity. The process in the layered structure shown in Fig. 1 is similar. The operating principle is as follows: when a portion of the light passes from the glass (refractive index: $n_{\mathrm{gl}})$ prism through the thin gold layer ($n_{\mathrm{Au}}$) and propagates in the polymer spacer layer ($n_{s}$), the propagation angle is such that the wave is totally internally reflected at the spacer/water ($n_{w}$) interface. Therefore, the refractive indices of the structure must satisfy the following relationship.

(2) $$n_{w}<n_{s}<n_{\mathrm{gl}}$$

When the refractive index of water changes, a change also occurs in the phase of the reflection coefficient at the spacer/water interface, and this causes a change in the cavity resonance conditions, as expressed by Eq. (1). Similar to the SPR case, a change in the resonance conditions is converted into a change in the reflectivity. The responsivity, which is defined as reflectivity change with respect to the acoustic pressure, ∆R/∆P, is further evaluated.

#### 2.2.1 Acousto-optic effect-based sensing mechanism

Considering, for the moment, the acousto-optic effect along, the factors that determine the responsivity are (i) the phase term $\phi_{1}$, because it is related to $n_{w}$ and determines the resonance dip, and (ii) the full width at half maximum (FWHM) of the reflectivity.

Analytical calculations readily show that for a light wave propagating at an angle θ in the spacer layer, where θ exceeds the critical angle for the spacer/water interface, the change in phase of the reflection coefficient $\Delta \phi_{1,s}$ with TE polarization (which generally provides the best response) and the water refractive index change $\Delta n_{w}$ are given by:

(3) $$\Delta \phi_{1,s}=\frac{2n_{w}n_{s}\cos\theta_{s}\Delta n_{w}}{\left(n_{s}^{2}-n_{w}^{2}\right)\sqrt{n_{s}^{2}\sin^{2}\theta_{s}-n_{w}^{2}}}$$

The derivation of this expression and its graphical representation are presented in Appendix 1. It can be shown using this expression that at angles above the critical angle, the phase change is greater with a smaller angle of incidence, although the acoustic bandwidth is smaller, as discussed in Appendix 1.

**Fig. 3:**
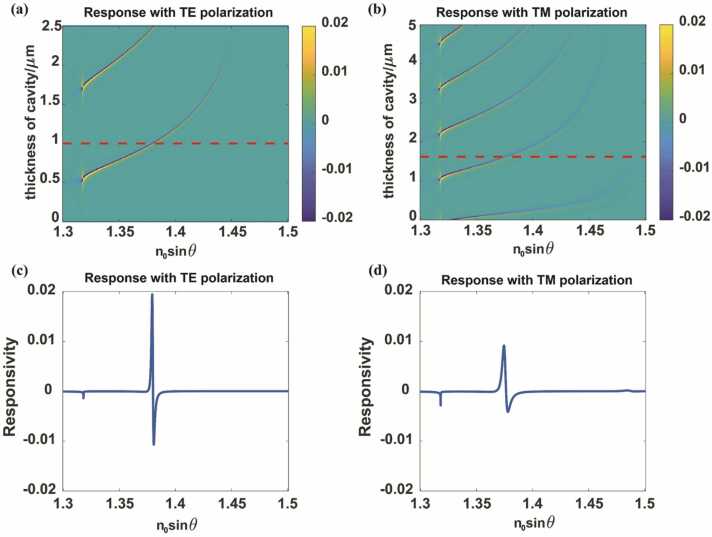
Response map showing variation of responsivity with $n_{0}\sin\theta$ and cavity thickness for (a) TE and (b) TM polarizations. (c) Response with a 1-µm-thick cavity and TE polarization. (d) Response with a 1.6-µm-thick cavity and TM polarization.

Consider the application of acoustic pressure of 1 MPa at the top surface, which results in a refractive index perturbation in the superstrate ($n_{w}$) that changes from 1.318 to 1.318135, with an acousto-optic coefficient of water of $∆n/∆p=1.35\times 10^{-10}{\mathrm{Pa}}^{-1}$. Fig. 3(a) and (b) show the response maps with respect to the cavity thickness and the incident wave vector ($n_{0}\sin\theta$) for the TE and TM polarizations, respectively. Fig. 3(c) and (d) show the responsivity with a cavity thickness of 1μm for TE polarization and that with a cavity thickness of 1.6μm for TM polarization, respectively. Fig. 3 shows that sensing with the TE polarization provides better response than sensing with the TM polarization. Furthermore, when using the same polarization, sensing with modes that are closer to the critical angle provides higher response. As shown in Fig. 2(c) and (d), the FWHM for the TE polarization is sharper than that for the TM polarization because the absorption loss for the TM polarization is always greater than that for the TE polarization when the gold layer thickness is the same; this is even more significant in the case here because the optimum thickness for the TE polarization (12 nm) is less than that for the TM polarization (30 nm) [35]. The TIR-FP can use its evanescent field as a detection probe, which is the same approach as the SPR. At 1550 nm, the penetration depth of the SPR is approximately 680 nm, whereas that of the TIR-FP is approximately 340 nm. This indicates that for acousto-optic effect-based sensing, the TIR-FP tends to have a greater bandwidth than the SPR, along with enhanced response, and when an appropriate system is used, the acoustic bandwidth can reach up to the gigahertz scale.

#### 2.2.2 Cavity length-based sensing mechanism

The resonant conditions in the cavity can also be perturbed by a change in the cavity length d. To calculate the change in the cavity dimensions, it is necessary to calculate the strain distribution throughout the cavity. If P(x) is used to represent the pressure distribution in the cavity and strain is given by ε(x), the total dimensional change in the cavity Δl is given by

(4) $$\Delta l=\int_{0}^{d}\varepsilon \left(x\right)\mathrm{dx}=\int_{0}^{d}\frac{P(x)}{M}\mathrm{dx}$$

where the polymer layer occupies the space between 0 and *d*, and *M* is the modulus for the axial deformation, which is $c_{11}$, because the layer is constrained from dimensional changes in the plane of incidence. Note here that the pressure and the strain are phasor quantities, and when the wavelength of the incident sound wave is comparable to the layer thickness *d*, significant variations will occur in the phase of the strain signal that will result in a reduction of the dimensional change in the cavity. This has the effect of reducing the ultrasonic transducer bandwidth. A similar effect also applied to the acousto-optic mechanism, but because the wave is evanescent in water, the change in the refractive index is only sensed over a small region in the water very close to the spacer/water interface. The bandwidth of the acousto-optic mechanism is therefore much greater than that associated with cavity deformation.

It is possible to calculate the pressure field numerically using a finite element method software package, e.g., COMSOL. However, the current arrangement lends itself to a simpler representation in terms of acoustic impedance.

**Fig. 4:**
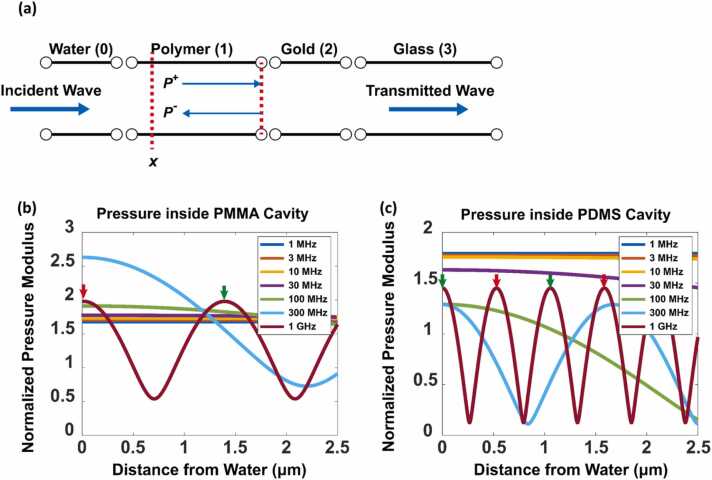
(a) Transfer matrix model of acoustics in the TIR-FP. Acoustic pressure distributions of (b) PMMA and (c) PDMS as cavity materials. The cavity-water interface is at 0 µm and the cavity-gold interface is at 2.5 µm. Blue, orange, yellow, violet, green, cyan, and brown represent input frequencies of 1 MHz, 3 MHz, 10 MHz, 30 MHz, 100 MHz, 300 MHz, and 1 GHz, respectively.

The acoustic impedance is given by the product of the velocity and material density, and this impedance can be used to calculate the acoustic reflection and transmission coefficients from a layered structure, such as the TIR-FP shown in Fig. 4(a) [36]. Using the transmission coefficient, one can then backpropagate to the interface between the gold surface and the spacer. This then gives forward and backward propagating waves, i.e., $P^{+}$ and $P^{-}$ at this interface. The total pressure at any point is simply given by the sum of these two complex quantities multiplied with propagation phase factors. The calculation process is presented in detail in Appendix 2.

PMMA and PDMS were considered for use as the FP cavity materials because of their outstanding optical transparency and mechanical properties. The properties of these materials are listed in Table 1. The acoustic pressure inside cavity layers made from PMMA and PDMS are shown in Fig. 4(b) and (c), respectively. The gold layer is very thin, it has little impact on the acoustic performance of the device, but it is included for completeness here. Peaks marked in green and red indicate the phase cancellation maxima and minima.

**Table 1:** Optical and mechanical properties of PMMA and PDMS.

	PMMA	PDMS
Refractive Index n	1.4958@532 nm	1.3976@532 nm
	1.4771@1550 nm	1.3804@1550 nm
Density ρ	1180 kg/m^3^	965 kg/m^3^
Sound Velocity (longitudinal) $c_{l}$	2757 m/s	1076.5 m/s
Bulk Modulus M	8.97 GPa	1.08 GPa

**Fig. 5:**
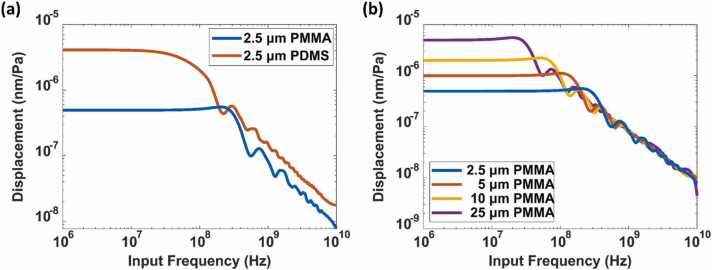
(a) Displacement per Pa vs. input frequency for 2.5 µm PDMS and PMMA layers. (b) Displacement per Pa vs. input frequency for PDMS layers of 2.5 µm, 5 µm,.

At low frequencies, when compared with the acoustic wavelengths, the spacer layer thickness (2.5 μm) is small, and thus the acoustic field is uniform. However, when the frequency increases, the wavelength decreases, and the total acoustic field stored inside the cavity eventually decreases until $\lambda \ll d_{\mathrm{cavity}}$. Summing of the total acoustic field and dividing it by the modulus gives the total displacement per pascal at each frequency, as shown in Fig. 5. Within the bandwidth, the PDMS cavity provides a displacement of $4.0455\times 10^{-6}$ nm Pa^−1^, whereas the PMMA cavity gives $4.9465\times 10^{-7}$nm Pa^−1^. Additionally, as shown in Fig. 5(a), the PMMA cavity gives a 3 dB bandwidth of 390 MHz, whereas the PDMS cavity gives a 3 dB bandwidth of 100 MHz for the same mechanism*.* Furthermore, the trade-off between the bandwidth and the displacement performance is demonstrated in Fig. 5(b). When the thickness increases by one order to 25 µm, the displacement increases to $4.0455\times 10^{-5}$ nm Pa^−1^, whereas the bandwidth narrows to less than 40 MHz.

Combining optical Fresnel calculations with calculation of displacement. Since PDMS provides 10 times more displacement than PMMA, the responsivity of PMMA is $3.117\times 10^{-7}{\mathrm{Pa}}^{-1}$ and that of PDMS is $3.043\times 10^{-6}{\mathrm{Pa}}^{-1}$.

SPR based sensors have been used in many photoacoustic sensing and imaging applications as optical detection probes [20], [37]. Theoretically, a conventional SPR produces a response of $3\times 10^{-8}$ ${\mathrm{Pa}}^{-1}$. Therefore, the TIR-FP structures with PMMA and PDMS improve the performance by orders of magnitude over conventional refractive index sensing mechanism when using the same excitation and detection configuration, and thus can provide an immediate upgrade for ultrasound and photoacoustic sensing and imaging. It should be noted that because mechanical properties such as the sound velocity are mostly frequency-dependent, and there have not been complete records of these properties for PMMA and PDMS in the literature, there is thus some uncertainty in the predictions due to the variability in the precise acoustic properties.

In summary, at low acoustic frequencies, the change in the cavity length is the dominant mechanism for acoustic sensing, this is particularly the case for the highly compliant PDMS layers. Thicker layers will be more sensitive but have lower bandwidth. As the wavelength of the acoustic wave approaches that of the cavity dimensions there is a fall-off in the response due to the change in the cavity length as the localized displacement throughout the cavity no longer adds in phase, so the total displacement decreases. In this case, the change in the refractive index of the fluid becomes dominant, where the bandwidth limiting dimension is the extent of the optical evanescent field in the liquid. To get the very highest bandwidth a shorter interrogation wavelength improves the confinement of the evanescent field. Furthermore, for high bandwidth applications, PMMA is preferable to PDMS in the sense that it will support more obliquely incident light which, in turn, gives a more confined evanescent field. These issues are discussed in more detail in Appendix 2.

## 3 Experimental results

### 3.1 Experimental setup

**Fig. 6:**
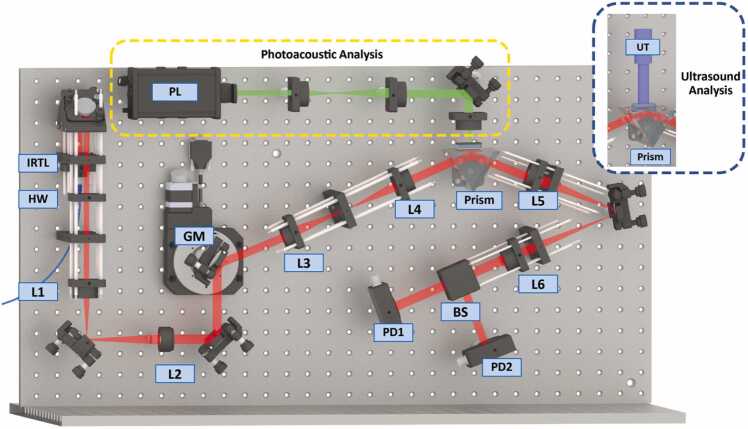
Experimental setup. IRTL: infrared tunable laser; HW: half waveplate; GM: galvomirror; PL: nanosecond pulse laser; UT: ultrasound transducer; BS: beam splitter; PD: photodetector.

Optical resonance of the TIR-FP structure features narrow linewidths in terms of both the wavelength and the angular domain. To validate the transducer performance a system that is capable of both angular and wavelength interrogation was constructed as shown in Fig. 6. Wavelength interrogation was realized using a fiber-coupled linearly polarized infrared tunable laser (TSL-710, Santec, Japan) that was centered at 1550 nm and tunable from 1480 nm to 1620 nm. The beam polarization was controlled using an achromatic half waveplate (AHWP10M-1600, Thorlabs, USA). The beam was then expanded using lenses L1 (*f*=100 mm, Thorlabs, USA) and L2 (*f*=150 mm, Thorlabs, USA) and projected onto a customized galvomirror, e.g., a motorized scanning mirror (PF10–03-P01 mounted on an HDR50/M stage; Thorlabs, USA) for the angular interrogation function. The beam was then projected onto the sample above the equilateral prism (OPRE32–22, Zolix, China) and collected using two photodetectors. The first detector (PDF10C2, Thorlabs, USA), which had a bandwidth ranging from DC to 20 Hz, was designed to monitor and compensate for power drift of the laser, whereas the second photodetector (PDA05CF2, Thorlabs, USA), which had a bandwidth of 150 MHz, was used for data acquisition and was connected to a low noise amplifier with a bandwidth of 150 MHz and then to a computer-controlled oscilloscope (MSOS254A, Keysight, USA) with a fixed bandwidth window of 500 MHz. The system can support both ultrasound and photoacoustic signals for responsivity and bandwidth analysis, respectively.

For the responsivity investigation, the ultrasound signal was generated using an ultrasound transducer (UT) as shown in blue dashed inset of Fig. 6 with a center frequency of 15 MHz that was excited using electric pulses with 16 different amplitudes from a pulse generator (DPR300, JSR, USA). The transducers and the pulse generator were calibrated using a hydrophone (HMB-200, Onda, USA). The amplitude levels correspond to acoustic pressures between 129 and 585 kPa. For the bandwidth investigation the ultrasound transducer was replaced with photoacoustic excitation, here a fast laser pulse (Nd:YAG laser operating at 532 nm (532 nm, Changguang, China) was focused onto a graphene layer with a thickness of approximately 100 nm to create ultrasound, this produced wide-band acoustic pulses as shown in the yellow dashed area in Fig. 6. These pulses were then detected using the designed sensor. The graphene layer was selected as the absorber to convert the laser intensity into acoustic waves because of its stiffness and high absorption. These two properties affect the bandwidth and the amplitude of the generated acoustic signals.

A conventional SPR sample with 30-nm-thick Au layer was tested experimentally as the reference of responsivity and bandwidth performance. The reason for this arrangement is so that the comparison can be made within the exactly the same optical system, so they reference our system to a relatively well-known and easily replicated structure. The measurements of the SPR sample give a responsivity of $1.517\times 10^{-8}{\mathrm{Pa}}^{-1}$as presented in Appendix 4, which generally agrees with the results cited in other literatures [12], [31]. The SPR sample also serve a role as a reference for adjustment of the polarization by minimizing reflectivity and calibration of the absolute angle since SPR requires a fixed angle of 63.60° with TM polarization component.

### 3.2 Total-internal-reflection-based Fabry–Pérot structure

The TIR-FP modes discussed here can be excited using both TM and TE polarizations. However, for the same gold layer thickness, the TE polarization tends to show a smaller loss than the TM polarization, thus resulting in a resonance with a narrower FWHM. Given the same changes in refractive index or cavity thickness, the TE modes provide a better response. Therefore, the experimental studies of the designed TIR-FP structure were conducted using the TE polarization.

Two batches of devices were fabricated with PMMA and PDMS as their FP cavity materials. Each material was spin-coated on top of a 12 nm thick sputtered gold layer. The 12 nm thickness was the optimum operating thickness determined for the device with the TE polarization based on consideration of both the strength of the resonance and the responsivity. The thicknesses of the PMMA layer and the PDMS layer were measured using a surface profiler to be 1.9 µm and 3.4 µm, respectively. In the experiment, the wavelength was first fixed at 1550 nm, and the angle of incidence was initially adjusted to be at the resonance dip. Then, the wavelength was fine-tuned toward the maximum responsivity point.

**Fig. 7:**
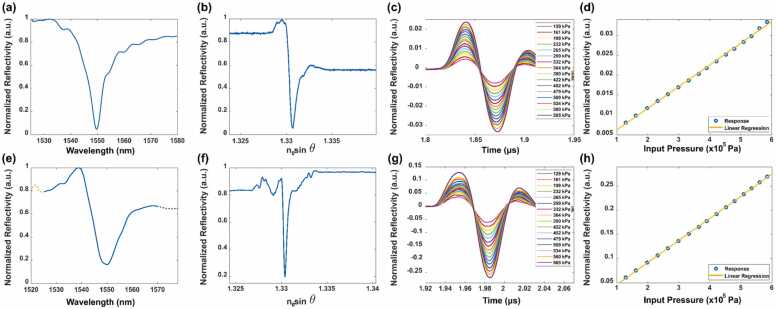
Results for PMMA- and PDMS-based TIR-FPs. Reflectivity of PMMA measured vs. (a) wavelength and (b) angular scanning profiles at 1550 nm, (c) response of 15 MHz UT to various input acoustic pressures, and (d) maximum recorded responses vs. the.

Fig. 7(a) and (b) depict the wavelength scanning and angular scanning profiles of the FP resonance when using the PMMA layer as the resonant cavity. As measured from Fig. 7(a), the resonance of the PMMA-based structure has an FWHM of 8.68 nm in terms of wavelength and $9.5\times 10^{-4}$ in terms of $n_{0}\sin\theta$. The temporal responses of the 15 MHz UT to 16 amplitude levels are shown in Fig. 7(c). Fig. 7(d) shows the peak values recorded for each of the amplitude levels plotted vs. the incident acoustic pressure with excellent linearity ($R^{2}=0.9999$). The responsivity is $5.45\times 10^{-8}{\mathrm{Pa}}^{-1}$, which represents a 3.6-fold improvement when compared with the SPR sensor measured in the identical system.

**Fig. 8:**
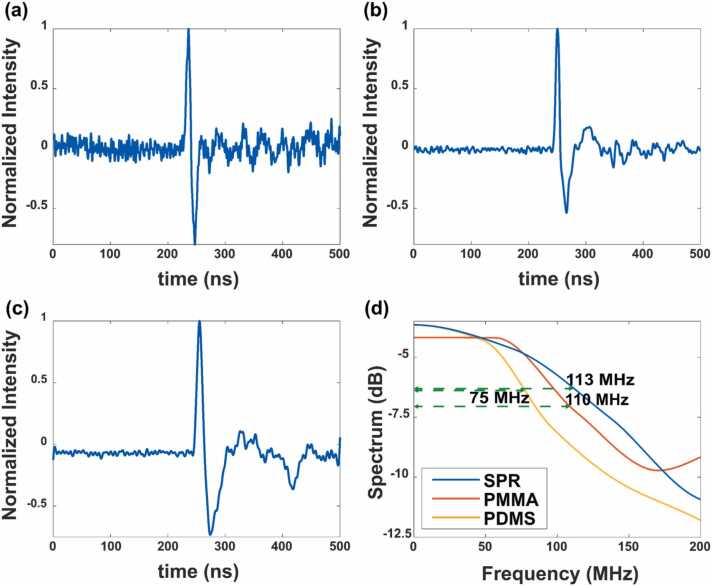
Photoacoustic results for the (a) SPR, (b) PMMA-, and (c) PDMS-based TIR-FPs and (d) their FFTs for the bandwidth analysis.

For the PDMS structure, as shown in Fig. 7(e) and (f), the FWHM is 12.5 nm in terms of wavelength and $3.8\times 10^{-4}$ in terms of $n_{0}\sin\theta$. These results are wider in wavelength terms and narrower in terms of $n_{0}\sin\theta$ when compared with PMMA. For improved display of the results, a Savitzky-Golay filter was applied to the wavelength and angular profiles for both PMMA and PDMS. For the angular profiles, the main noise source was random noise. For the wavelength profiles, there were interference sources from the optical system that overlapped constructively with the resonance. Therefore, the FWHM values in Fig. 7(a) and (e) may be smaller than the corresponding simulation results. As shown in Fig. 7(e), the resonance dip for PDMS was shallower when compared with the dips for PMMA and the SPR. This can be explained based on the recent reports claiming that PDMS has a small imaginary component in its refractive index at 1550 nm [34]. As shown in Fig. 8(g) and (h), the PDMS sample produced a responsivity of $4.5\times 10^{-7}{\mathrm{Pa}}^{-1}$ at 15 MHz, representing a 30-fold improvement over the conventional devices. For a conventional FP structure with a 20-µm thick polyethylene terephthalate (PET) layer sandwiched between 10 nm and 100 nm gold layers, the typical responsivity is around $4.6\times 10^{-7}{\mathrm{Pa}}^{-1}$ [12]. This result is comparable with that of the proposed PDMS-based TIR-FP although the bandwidth of our system will be much greater.

### 3.3 Photoacoustic sensing

**Fig. 9:**
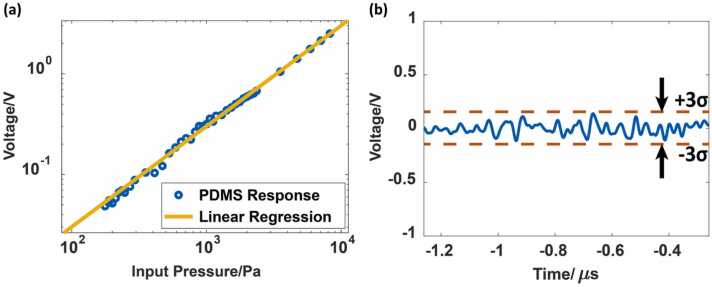
(a) Response of PDMS based TIR-FP with a function generator as the driver of the ultrasound transducer. The data is marked in blue dots and its linear regression is marked in a yellow line. (b) Single-shot noise level recorded before ultrasound trigger.

Responsivity and bandwidth are two key parameters in acoustic sensor characterization and applications. However, commercially available piezoelectric UTs usually have narrow bandwidths and are therefore unsuitable for use in characterization of the proposed structure. Therefore, the UT was replaced with a high-energy nanosecond laser focused on a graphene layer that was in liquid contact with the structure. Broadband photoacoustic signals can then be generated by the layer and detected using the proposed photoacoustic sensor. A spectrum comparison of the generated signal with the acquired signal gives the bandwidth information for the proposed structure. Fig. 9(a)-(c) show the temporal responses of the photoacoustic signals for the SPR, PMMA, and PDMS samples, respectively. Fast Fourier transforms (FFTs) of these three signals are shown in Fig. 8(d). As measured from the FFT, the acquired bandwidths for the SPR, PMMA, and PDMS-based TIR-FPs are 113 MHz, 110 MHz, and 75 MHz, respectively. The received signal bandwidth for PMMA is limited by the pulse width of the excitation laser, the stiffness of the absorbing material, and the bandwidth of the photodetector. The pulse width of the visible excitation laser at 532 nm is 3.4 ns, as measured using a visible fast photodetector. The 3 dB bandwidth of the amplified photodetector (PDA05CF2, Thorlabs, USA) is 150 MHz. The bandwidth of the PDMS is consistent with the theoretical considerations in Section 2.2.2 where a 2.5 µm predicted a 100 MHz bandwidth scaling this value with the measured thickness gives a bandwidth of 74 MHz. These measurements demonstrated the capability of the proposed device for photoacoustic sensing and its potential in photoacoustic microscopy and imaging.

It is worth noting that the absorber used in the experiment was a graphene layer, which does not give complete absorption of incident pulsed light. And there is light leakage that continues to propagate onto the TIR-FP transducer, causing a thermal signal with a delay of tens of nanoseconds from the trigger. However, the thermal signal lasts no more than 100 nanoseconds and does not overlap with the photoacoustic signal. By changing the propagation distance between the graphene absorber and the transducer, the timing of the investigated signal was consistent with a sound wave propagating through the liquid layer. Therefore, it was confirmed that the measured signals were photoacoustic signals.

### 3.4 Sensitivity analysis

By extrapolating the responsivity down to the noise level, we can then obtain an evaluation of the detection limits of the devices, i.e., the noise equivalent pressure (NEP) with a SNR of 1. A small NEP corresponds to more precise measurement and enables use of low laser power and therefore provides greater safety for the subject investigated. In order to better reflect the noise equivalent level, a series of detailed responsivity measurement have been made with the input acoustic pressure in the regime of < 1 kPa with a fixed bandwidth of 20 MHz on the oscilloscope. In this measurement, a function generator (33622 A, Keysight, US) was utilized to generate a pulse of various amplitudes and drive the ultrasound transducer to generate the mentioned small ultrasound signals. The ultrasound transducer with the function generator was calibrated with the hydrophone. Response maximum of the PDMS based TIR-FP is plotted against each incident ultrasound pressure as shown in Fig. 9(a). The results demonstrate excellent linearity ${(R}^{2}=0.9985)$ of the TIR-FP with small input signals. The response of the resonator is about 301.75 mV per 1 kPa. Please note that, to better eliminating the effect of the noise and more precise examination of the linearity of the response of the designed TIR-FP, 9-time averaging was used in the measurement. This gives a 3-fold improvement in signal-to-noise ratio. However, in the measurement of the noise levels, no averaging was used.

Then noise levels were recorded from − 1.26 μs to − 0.26 μs, which is before the pulse was generated, and measured to be 48.47 mV as shown in Fig. 9(b). Therefore, the NEP of the system is about 160 Pa. The main noise type in the system was additive Gaussian noise since no obvious increase in NEP was observed when increasing the gain of the amplifier.

## 4 Conclusion

In this paper, we have proposed a photoacoustic sensor composed of a total-internal-reflection-based FP structure, along with a theoretical analysis and experimental demonstration of this device. The structure uses one reflection layer rather than the two used in conventional FP structures, which greatly reduces the acoustic impedance mismatch created by the metal or dielectric layers that are conventionally used. The structure is investigated in intensity-interrogation based system with Kretschmann configuration for investigation above the critical angle, which is the same as that used for optical surface wave based optical sensors [31], [38]. An SPR based sample have also been tested experimentally as a benchmark and reference. The proposed PMMA- and PDMS-based TIR-FP structures achieved responsivities of $5.45\times 10^{-8}{\mathrm{Pa}}^{-1}$ and $4.5\times 10^{-7}{\mathrm{Pa}}^{-1}$, representing 3.6- and 30-fold improvements over the conventional SPR sample measured within the identical system, respectively. As a device that can be excited using the same configuration as the conventional SPR, the proposed structure demonstrated its potential with a 30-fold improvement in its acoustic sensing capability combined with a simple layout and easy fabrication. In addition, the proposed structure uses a thin FP cavity with a thickness of less than 4 µm that circumvents the trade-off between bandwidth and responsivity which is faced by conventional FP resonators. Investigations with broadband photoacoustic signals showed that the proposed structures have estimated bandwidths of 110 MHz and 75 MHz for PMMA and PDMS, respectively. At wavelengths around 1550 nm, the optimum gold layer thickness for the TE polarization is 12 nm, and the corresponding thickness for the TM polarization is 30 nm. For the TE polarization, the structure uses a thin gold layer with a thickness of 12 nm that provides better optical transparency at visible wavelengths, thus allowing potential integration with other imaging modalities. The PMMA-based TIR-FP struck a balance between responsivity and bandwidth. A bandwidth of 110 MHz was measured for this structure with broadband photoacoustic signals. This also represents a demonstration of the structure’s potential for future applications. For the PDMS layer, sensitivity of 160 Pa over a bandwidth of 20 MHz was observed in the current intensity-interrogation based system. Compared with the conventional FP transducers, the proposed TIR-FP sensor consists of only two layers, therefore, the fabrication complexity is greatly reduced, and the yield can be improved. TIR-FP can therefore be a cost-efficient and performance-enhanced choice for photoacoustic sensing and imaging [9].

Future improvements can be made to enhance the sensitivity and bandwidth. As previously discussed, the NEP-based sensitivity is related to both SNR of the system and the responsivity of the transducer. Since the current bandwidth of the transducers stands at 75 MHz and 110 MHz, with a thicker spacer layer, the NEP performance can be projected to go well below 50 Pa within the same interrogation system while retaining a relatively large bandwidth (>20 MHz) [26]. Sensitivity can be further improved using phase interrogation and an additional arm for cancellation of common mode noise, where improvements of a factor of at least 4 can be expected [15], [39]. The bandwidth can be further improved by using narrower laser pulse widths and photodetectors with wider detection bandwidths. Further fine-tuning between responsivity and bandwidth can be achieved via fine-tuning of the FP cavity thickness. As a more durable material than PMMA, parylene may potentially be a better choice for the cavity when producing the sample structures in bulk volume [11], [40].

## Funding

Mengqi Shen acknowledges the support of the 10.13039/501100001809National Natural Science Foundation of China [Grant number 62205218].

## Declaration of Competing Interest

The authors declare that they have no known competing financial interests or personal relationships that could have appeared to influence the work reported in this paper.

## Data Availability

Data will be made available on request.
