# Supplementary material for: A total-internal-reflection-based Fabry–Pérot resonator for ultra-sensitive wideband ultrasound and photoacoustic applications
Source: Photoacoustics. 2023 Feb 24;30:100466. doi: 10.1016/j.pacs.2023.100466 (PMC10011501; doi:10.1016/j.pacs.2023.100466)
Supplement: Supplementary file 1 — Supplementary material [file mmc1.docx]

# Appendix 1. Analytical expression for the phase shift at the spacer-water interface

It is well known that the resonant conditions for the SPR will vary as the refractive index of the final medium changes. A similar effect occurs at the interface of the polymer and the liquid. Since we are looking at the effect of refractive change in the liquid, we may assume that the dimensions of the resonator do not change, and the effects of dimensional change may be considered independently. The acousto-optic mechanism changes the index of the water which, in turn, changes the phase of the reflection coefficient, at the water/polymer. The phase change induced by this refractive index change perturbs the resonant conditions that were expressed in Eq. (1). This is shown in Fig. A1.

The phase change of the reflection coefficient may be calculated analytically. We concentrate on the TE polarization because it provides the best sensitivity, but the case for the TM polarization runs along similar lines. The optical reflection coefficient at the layer/water interface for the TE polarization is given by:

$r_{s}=\frac{n_{s}\cos\theta_{s}-n_{w}\cos\theta_{w}}{n_{s}\cos\theta_{s}+n_{w}\cos\theta_{w}}$ (A1.1)

where the subscripts *s* and *w* refer to the polymer spacer layer and the water, respectively. By combining this with Snell’s Law and noting that the incident wave in the layer is incident at an angle above the critical angle, we obtain:

$\cos\theta_{w}=i\sqrt{\left( \frac{n_{l}\sin\theta_{l}}{n_{w}} \right)^{2}-1}$ (A1.2)

This means that in Eq. (A1.1), the first term in the numerator is real and the second term is imaginary. The numerator and denominator are thus complex conjugates of each other, which means that we can calculate the phase change $\phi$ in the numerator and then double it to obtain the overall phase change of the reflection coefficient, $2\phi$.

We then take the inverse tangent of the imaginary part of the denominator with respect to the real part and differentiate with respect to $n_{w}$ to obtain the phase change caused by the change in the refractive index of the water $\Delta n_{w}$ taken as $1\times{10}^{-4}$ RIU:

$2\Delta\phi=2\frac{d\phi}{dn_{w}}\Delta n_{w}=\frac{2n_{w}n_{l}\cos\theta_{l}\Delta n_{w}}{\left( n_{l}^{2}-n_{w}^{2} \right)\sqrt{n_{l}^{2}\sin^{2} \theta_{l}-n_{w}^{2}}}$

(A1.3)

The analytical result calculated using Eq. (A1.3) is shown in Fig. A1.


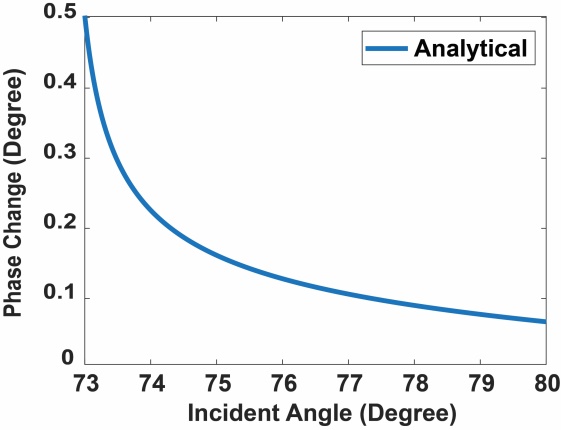


Fig. A1. Phase change calculated using the analytical expression in Eq. (A1.3) and the optical parameters associated with PDMS. Note that the incident angles on the *x*-axis refer to the incident angle in the spacer not the glass prism.

The figure shows that the sensitivity decreases as the angle of incidence increases above the critical angle of spacer/water interface, i.e., 72.7°. In contrast, the penetration depth decreases as the angle of incidence increases, which means that the decreasing sensitivity corresponds to an increase in the bandwidth as discussed in detail in Appendix 3. The 1/e penetration depths at 1550 nm for the squared field are 1625 nm and 372 nm at 73° and 80°, respectively.

# Appendix 2. Calculation of the pressure distribution and the associated strain in the spacer layer

There are several different ways to perform this calculation, but the approach that uses the acoustic impedance is both straightforward and highly computationally efficient. This method is essentially that provided by Azzam and Bashara [40], but with a slight extension to allow the fields to be obtained at arbitrary positions.

Since the acoustic waves travel along the structure and hit all interfaces, normally any obliquity factors can be omitted. In that case, the acoustic impedance $Z$ of a material is simply the product of its density $\rho$ and the sound velocity $v$ in the material:

$Z=\rho v$ (A2.1)

Fig. 4(a) shows the transmission line structure of our transducer, where the different material layers have been given numerical labels as shown. We can give the transfer matrix that relates the input pressure wave from the liquid to the transmitted wave in the glass as follows:

$\left[ \begin{matrix} P_{0}^{+} \\ P_{0}^{-} \end{matrix} \right]=\left[ \boldsymbol{S}_{\boldsymbol{tot}} \right]\left[ \begin{matrix} P_{3}^{+} \\ P_{3}^{-} \end{matrix} \right]$ (A2.2)

where $\boldsymbol{S}_{\boldsymbol{tot}}$ represents the field propagation through the structure.

$\boldsymbol{S}_{tot}=\boldsymbol{I}_{01}\Phi_{1}\boldsymbol{I}_{12}\boldsymbol{\Phi}_{2}\boldsymbol{I}_{23}$ (A2.3)

In Eqs. (A2.2) and (A2.3), the subscripts refer to the layer numbers, and $I_{ij}$ refers to the transfer matrix across the *ij* interface when passing from the *j^th^* layer to the *i^th^* layer. This matrix is given in terms of the impedances of the *i^th^* and *j^th^* layers as:

$\boldsymbol{I}_{\boldsymbol{ij}}=\frac{1}{2Z_{j}}\left[ \begin{matrix} Z_{j}-Z_{i} & Z_{j}+Z_{i} \\ Z_{j}+Z_{i} & Z_{j}-Z_{i} \end{matrix} \right]$ (A2.4)

and the propagation matrix $\boldsymbol{\Phi}$ is:

$\boldsymbol{\Phi}_{\boldsymbol{i}}=\left[ \begin{matrix} \exp-ik_{i}d_{i} & 0 \\ 0 & \exp ik_{i}d_{i} \end{matrix} \right]$ (A2.5)

where *k_i_* and *d_i_* are the propagation constant and the thickness of the *i^th^* layer, respectively. The value of $P_{3}^{-}$ in Eq. (A2.2) is zero, because the medium is considered to be semi-infinite.

From this, we have

$P_{3}^{+}=P_{0}^{+}/\boldsymbol{S}_{\boldsymbol{tot}}(1,2)$ (A2.6)

Once this is known, we can then propagate the field toward the spacer/gold interface (as indicated by the dashed red layer in Fig. 4(a) to obtain the pressure field at this interface, which is given by:

$\boldsymbol{I}_{12}\boldsymbol{\Phi}_{2}\boldsymbol{I}_{23}\left[ \begin{matrix} P_{3}^{+} \\ 0 \end{matrix} \right]$ (A2.7)

The forward and backward propagating waves in the spacer layer are then determined simply by multiplying this pressure field by a propagation matrix in which the distance from the interface *x* shown in Fig. 4(a) replaces *d_i_* in Eq. (A2.5).

Summing the forward and backward propagating pressure waves then gives the total pressure at a specific position, and this pressure can be converted into a strain using Eq. (2). The forms of these propagation matrices show that the thickness of the polymer spacer layer should be much less than half of the acoustic wavelength to prevent phase cancellation and avoid a reduction in the responsivity.

# Appendix 3. Analysis of the bandwidth of refractive index sensing mechanism and its relation to the incident angle

The bandwidth due to the perturbation of the refractive index may be determined considering the situation depicted in Fig. 1(b). In this case there is an evanescent field shown by the red curve that emerges from the polymer spacer layer. The extent of this field depends on the wavelength of the incident light as well as the incident angle of the light in the polymer. If the angle is just above the critical angle, the field will extend deep into the liquid, whereas, if the angle is well above the critical angle, the field will be tightly confined. The variation of the refractive index in the liquid due to the pressure perturbation of the acoustic wave is represented by the grey shading. If the wavelength of the acoustic field is considerably greater than the decay length of the acoustic field, the light will experience an almost constant refractive index, whereas when the sound frequency is increased different parts of the evanescent field will experience different phase of the refractive index perturbation so that the response will roll off. Since the extent of the evanescent field is generally smaller than the cavity dimensions, the bandwidth due to this mechanism will generally be greater than that due to deformation of the cavity.

In order to estimate the bandwidth, we consider two quadrature phases of the refractive index variation in the water due to the acousto-elastic effect.

$n\left( x \right)=n_{0}+\Delta n\cos(k_{ac}x)$ (A3.1)

$n\left( x \right)=n_{0}+\Delta n\sin(k_{ac}x)$ (A3.2)

where $n_{0}$ is the unperturbed refractive index, $\Delta n$ is the maximum change in pressure for the give acoustic pressure, $k_{ac}$ is the wave vector of the acoustic wave and $x$ is the distance from the polymer/water interface.

The refractive index changes are converted to an impedance change which is propagated back to the polymer/water interface. This is simply performed by propagating the impedance piecewise through extremely thin layers as depicted schematically in Fig. 4(a), We then find the modulus of the impedance change projected onto the interface, this is the perturbation seen by the cavity.


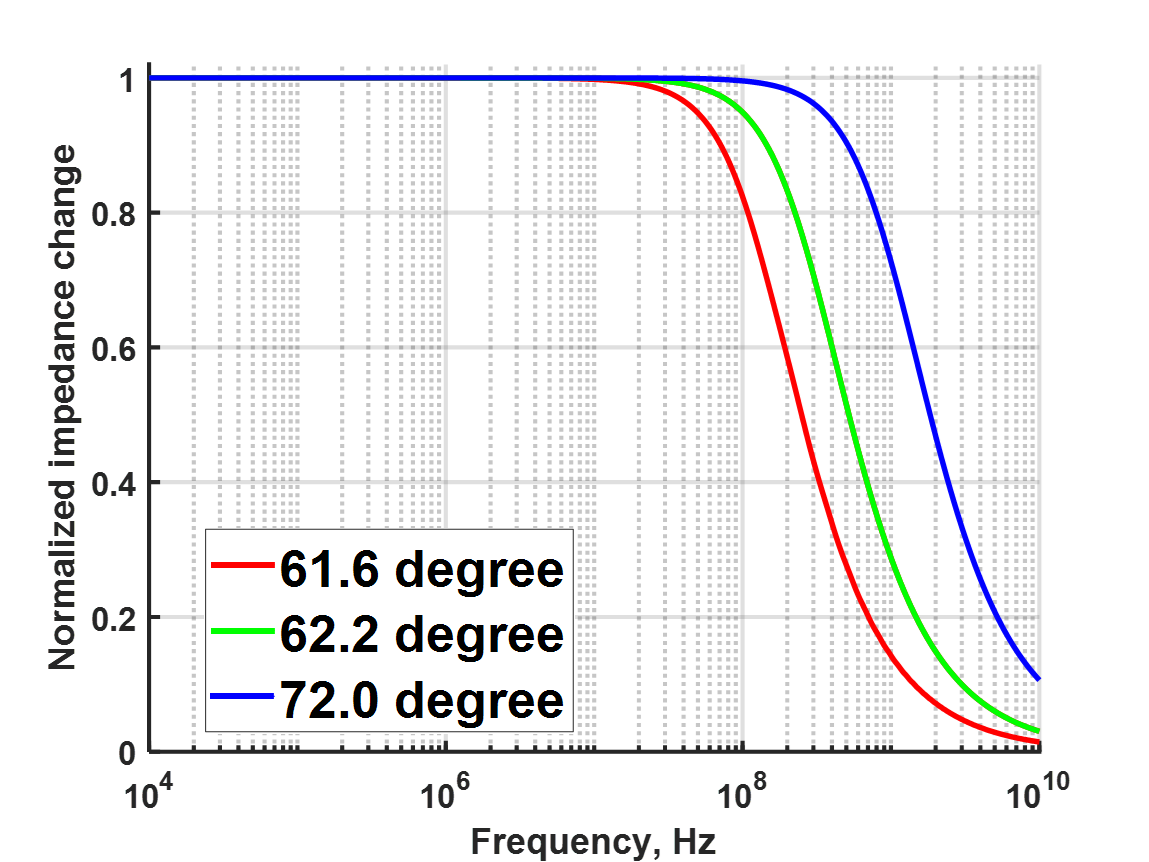


Fig. A2. Frequency response of normalized impedance change considering TIR-FP modes at various incident angles above the critical angle.

The results presented in Fig. A2 show the effect of changing the incident angle on the bandwidth of the response. The red curve corresponds to 61.6°, which is just above the critical angle, the impedance change falls to half the DC value at a frequency of about 250 MHz. If the incident angle of the PDMS layer is close to the SPR shown as the green curve at 62.2°, the half value point occurs at approximately 500 MHz. If we consider a large incident angle of 72° from the glass (blue curve), the bandwidth increases to about 2 GHz. Note that this angle of incidence cannot be achieved in a PDMS layer as the wave is not propagating at this incident angle, it is, however, entirely realizable in PMMA. Note that reducing the incident wavelength to the visible will enhance the bandwidth in proportion to the wavelength reduction.

# Appendix 4. Results of the SPR measured within the same system for reference

The results from the conventional SPR sample are presented for reference with respect to the proposed devices, as depicted in Fig. A3. The angular profile of the SPR sensor is shown in Fig. A3(a). The FWHM of the dip was $1.08\times{10}^{-2}$ in $n_{0}\sin\theta$ (equivalent to 0.89°), as shown in Fig. A3(a). Fig. A3(b) shows the temporal response acquired with the 15 MHz ultrasound transducer. Fig. A3(c) depicts the normalized intensity with respect to incident acoustic pressure and its linear regression. The slope of the linear fitting marks the responsivity of the SPR transducer, which is $1.517\times{10}^{-8} \mathrm{Pa}^{-1}$. The result shows excellent linearity with $R^{2}$ of 0.9985. The value agrees well with the maximum responsivity of $1.63\times{10}^{-8} \mathrm{Pa}^{-1}$reported in Ref. [12, 31].


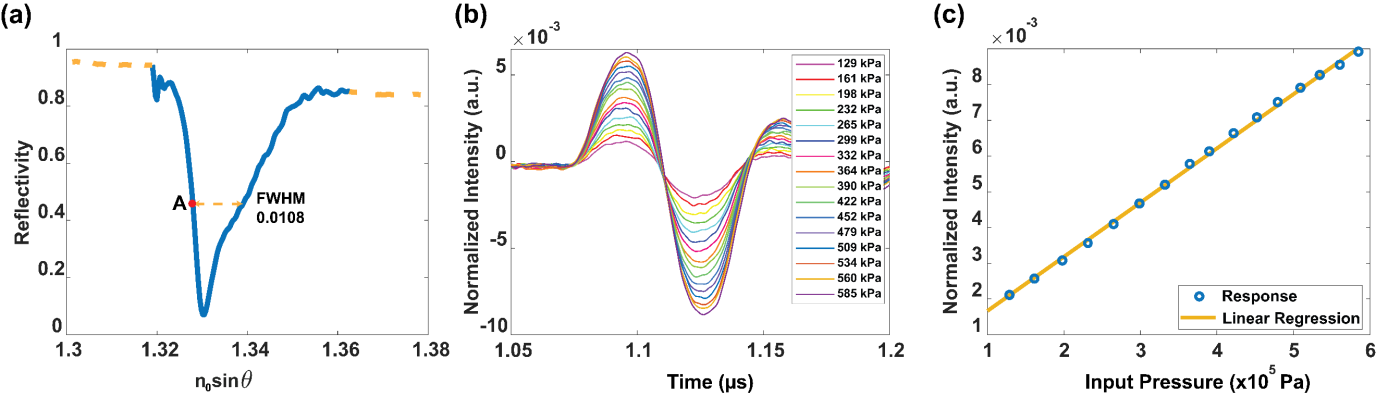


Fig. A3. Results for SPR sensor. (a) Reflectivity measured via angle scanning, (b) responses of 15 MHz ultrasound transducers to various input acoustic pressures, and (c) maximum recorded responses with respect to the input acoustic pressures.
